# Evaluation of Genetic Diversity and Structure of Turkish Water Buffalo Population by Using 20 Microsatellite Markers

**DOI:** 10.3390/ani11041067

**Published:** 2021-04-09

**Authors:** Emel Özkan Ünal, Raziye Işık, Ayşe Şen, Elif Geyik Kuş, Mehmet İhsan Soysal

**Affiliations:** 1 Department of Animal Science, Tekirdağ Namık Kemal University, 59030 Tekirdağ, Turkey; aysesen@nku.edu.tr; 2 Department of Agricultural Biotechnology, Tekirdağ Namık Kemal University, 59030 Tekirdağ, Turkey; risik@nku.edu.tr; 3 GenoMetri Biotechnology Research and Development Consultancy Services Limited Company, 35430 İzmir, Turkey; elif.geyik@genometri.com.tr

**Keywords:** Turkish water buffalo, microsatellite loci, genetic diversity, heterozygosity, genetic structure

## Abstract

**Simple Summary:**

In the present study, twenty microsatellite loci were tested to assess and analyze the genetic diversity between and within 17 different populations of Turkish water buffalo. The total number of animals sampled was 837, collected from six geographical regions: Marmara Region (MRM), Black Sea Region (BSR), Aegean Region (AER), Central Anatolia Region (CAR), Eastern Anatolia Region (EAR) and Southeastern Anatolia Region (SAR). All studied microsatellites markers showed allelic polymorphism. In this study, the results indicated a definite genetic diversity among the Turkish water buffalo populations which indicates the existence of at least two major clusters.

**Abstract:**

The present study was aimed to investigate the genetic diversity among 17 Turkish water buffalo populations. A total of 837 individuals from 17 provincial populations were genotyped, using 20 microsatellites markers. The microsatellite markers analyzed were highly polymorphic with a mean number of alleles of (7.28) ranging from 6 (ILSTS005) to 17 (ETH003). The mean observed and expected heterozygosity values across all polymorphic loci in all studied buffalo populations were 0.61 and 0.70, respectively. Observed heterozygosity varied from 0.55 (Bursa (BUR)) to 0.70 (Muş (MUS)). It was lower than expected heterozygosity in most of the populations indicating a deviation from Hardy–Weinberg equilibrium. The overall value for the polymorphic information content of noted microsatellite loci was 0.655, indicating their suitability for genetic diversity analysis in buffalo. The mean F_IS_ value was 0.091 and all loci were observed significantly deviated from Hardy–Weinberg Equilibrium (HWE), most likely based on non-random breeding. The 17 buffalo populations were genetically less diverse as indicated by a small mean F_ST_ value (0.032 ± 0.018). The analysis of molecular variance (AMOVA) analysis indicated that about 2% of the total genetic diversity was clarified by population distinctions and 88 percent corresponded to differences among individuals. The information produced by this study can be used to establish a base of national conservation and breeding strategy of water buffalo population in Turkey.

## 1. Introduction 

Water buffaloes have been reported to be of great importance to the lives of farmers and thus to the economies of many countries worldwide [1]. The number of water buffaloes in the world has increased rapidly over the past few decades, and according to Food and Agriculture Organization (FAO) statistics, there are about 208 million buffaloes in the world. Most of the world’s buffaloes live in Asia (96.79%), Africa (1.68%), the Americas (1.23%) and Europe (0.22%) [1,2].

According to 1974 FAO statistics, at that time, there was one million buffalo heads in Turkey. From 1984 to 1998, there has been a decrease in the buffalo breeding population of 75 percent, and the reason for this decrease in water buffaloes has been the preference for cattle breeding and increasing technology in agriculture over buffalos in the Aegean and Marmara Regions, where many buffaloes were found. In this period, all the improvement efforts for genotypes were only practiced on cattle in Turkey.

In Turkey, most farmers keep one or two buffaloes for family consumption, and this system is very widespread in villages, while farms with around 100 heads are located near the big cities. Despite the popular indifference, farming of this species has survived in order to promote productive systems in agreement with sustainable rural development and the trend to revalue autochthonous genetic types [3]. Thanks to the incentives ($100 for each female buffalo) introduced for water buffalo husbandry in recent years, the water buffalo population has risen to 178,397 heads [2]. Since 2011, the National Anatolian Water Buffalo Breeding Project is being implemented in Turkey by the Republic of Turkey Ministry of Agriculture and Forestry with the cooperation of different universities, research institutes and water-buffalo-breeder associations under the condition of breeders. Thus, with this project, the number of water buffaloes in Turkey has increased around 60,000 heads last decade due to economically support breeders that raised Anatolian water buffalos by the Republic of Turkey Ministry of Agriculture and Forestry [2]. Having over 70 different species under domestic and wild categories, domestic water buffaloes are divided into two groups, “River (Stream) Buffalo” and “Swamp Buffalo”. River buffalo with Indian origin is a race with a combined efficiency, mostly bred for meat and milk [4]. Swamp buffalo, known as “Carabao”, on the other hand, is not suitable for milk production and is a race found in China and Southeast Asia, and they are particularly used for meat production, rarely in milk production and being used as a draught animal [5].

Water buffaloes in Turkey originate from Mediterranean Water Buffaloes, a subgroup of river water buffaloes and are known as Anatolian Water Buffalo [3]. Turkey is composed of seven different geographical regions (Marmara, Aegean, Black Sea, Central Anatolia, Eastern Anatolia, Mediterranean and Southeastern Anatolia Regions). Buffaloes mainly reared at the Marmara Region (MRM), Aegean Region (AER), Black Sea Region (BSR), Central Anatolia Region (CAR), Eastern Anatolia Region (EAR) and Southeastern Anatolia Region (SAR) of Turkey [5]. The provinces with the highest amount of water buffalo presence are recorded as Samsun, Diyarbakır, Istanbul, Tokat, Bitlis, Muş, Afyon, Kayseri, Sivas and Amasya [2]. Buffalos are substantially raised for milk and meat production in Asian countries, Italy and Turkey. Buffalo milk that has a high percentage of fat is used for Turkish desserts and making special yogurt. This fatty part of buffalo milk is consumed in the traditional Turkish breakfast called “kaymak”. Moreover, buffalo meat is used in the Turkish sausage-making industry. Buffalos have high genetic diversity and the ability to adapt to harsh environmental factors [4,5,6]. Worldwide, the detection and conservation of genetic diversity in wildlife animals and local livestock breeds are the most important goals of researchers and breeders nowadays.

Genetic diversity can be detected with molecular markers, such as random amplified polymorphic DNA (RAPD), amplified fragment length polymorphism (AFLP), single nucleotide polymorphism (SNP) and, most commonly used, microsatellites [6,7,8], because microsatellites are highly polymorphic, co-dominant and neutral markers spread over the genome. Microsatellite markers or simple sequence repeats (SSRs) are widely used to expose genetic diversity in coding and non-coding regions of all prokaryotic and eukaryotic genomes [8,9,10]. Many genetic diversity studies have been carried out by using microsatellite loci in water buffalo populations in the world [8,9,10,11,12,13,14,15,16,17,18,19,20]. Researchers have revealed 14–61 polymorphic microsatellite markers in Egyptian [8], Indian [13,17,18] and Iranian [14] buffalo breeds. Ángel-Marín et al. [10] and Martinez et al. [15] have investigated the Colombian buffalo populations, using microsatellite markers. Zhang et al. [16] have revealed genetic divergence among swamp buffalo and river buffalo with microsatellite markers in China. Many studies have used microsatellite markers to describe the levels and the distribution of genetic diversity in water buffalo populations from different countries [8,9,10,11,12,13,14,15,16]; on the contrary, there are very few studies on genetic diversity in Turkish water buffalo populations [12,17,18] The genetic identification of Turkish water buffalos takes importance for the conservation of genetic diversity in indigenous breeds. The aims of this study were (I) to assess the genetic diversity within and between the Turkish water buffalo population; (II) to estimate the level of inbreeding, using 20 microsatellite loci; and (III) to identify the genetic relationship and describe geographical and genetic distinction between different water buffalo populations at different sites in Turkey.

## 2. Materials and Methods

This experiment was conducted at the National Anatolian Water Buffalo Breeding Project breeders buffalo in 6 different geographical regions in Turkey. The experimental protocol was approved by the Republic of Turkey Ministry of Agriculture and Forestry Pendik Veterinary Control Institute Local Ethics Committee (AEC approval number: 12/2013). This study was conducted between June 2013 and June 2015.

### 2.1. Sampling and DNA Extraction

In this study, a total of 837 blood samples from unrelated Turkish water buffalo (Bubalus bubalis) individuals were collected at random from 17 different populations located in 6 different geographical regions (17 cities, 37 districts and 119 villages) (Supplementary Figure S1). These regions have been chosen to represent the expected Turkish water buffalo populations. Most of these samples are local populations belonging to exceedingly small farms.

Blood samples were collected in vacutainer tubes that include EDTA (0.5 mM, pH 8.0) and stored at +4 °C until DNA extraction. Around 8 mL of blood per buffalo was taken, and the purification of genomic DNA was performed from 2 mL blood samples, using High Pure PCR Template Preparation Kit (Roche Molecular Systems Inc., Pleasanton, CA, USA), following the manufacturer’s protocol. All DNA extraction and polymerase chain reaction (PCR) amplification were performed by Geometry Biotechnology (http://www.genometri.com.tr/, accessed date 10 August 2020) in Istanbul.

### 2.2. Microsatellites Amplifications and Analysis

A total of 20 heterologous buffalo microsatellite loci were studied from a panel of 30 markers recommended by the International Society for Animal Genetics (ISAG) and Food and Agriculture Organization of the United Nations (FAO) working group for biodiversity study. The criterion for selection of the microsatellite loci was based on the high polymorphism information content value (PIC) and the number of exhibited alleles of the loci [12]. Information on the twenty-microsatellite investigated is presented in Supplementary Table S1. These microsatellites were analyzed to estimate various genetic diversity parameters. The microsatellite markers analysis was performed by using an Applied Biosystems 3130 Genetic Analyzer. The microsatellite markers were grouped in five sets of fluorescent-labeled primers. Five primer pairs were performed in each set for multiplex amplification. The forward primer for each locus was labeled with one of the four fluorescent dyes FAM, HEX and TAMRA (Applied Biosystems, Foster City, CA, USA) (Supplementary Table S1). The polymerase chain reaction analyses were carried out in a T100TM Thermal Cycler (Bio-Rad), using the primers listed in Supplementary Table S1. The reaction mixture was composed of genomic DNA (50 ng), 200 mM dNTPs, 2.0 mM MgCl_2_, 1X PCR buffer, 10 pmol forward and reverse primers and Taq DNA polymerase (0.5 u/sample). The PCR was performed with a total reaction volume of 25 µL, using the following thermal conditions, 94 °C for 10 min, followed by 32 cycles of 94 °C for 1 min, 55 °C for 30 s, 56 °C for 3 min and a final extension at 60 °C for 1 h. Amplified DNA was controlled in 1% agarose gel, using SYBR™ Safe DNA Gel Stain.

After agarose gel electrophoresis for 20 microsatellites, allele size was identified on all samples with an ABI Prism^®^ 3130 Genetic Analyzer (Applied Biosystems, Foster City, CA, USA), using the GeneScan^®^ Analysis Software (Applied Biosystems, Foster City, CA USA), which check different alleles via size comparison with standard DNA size markers GeneScan 500 ROX Dye (Applied Biosystems, Foster City, CA, USA). Allele sizes were calculated with the GeneMapper^®^Software V4.0 (Applied Biosystems™, Foster City, CA, USA)

### 2.3. Data Analysis

Allele frequencies, the total number of alleles, the mean number of alleles (N_a_), the number of effective alleles (N_e_), allelic richness (R_S_), polymorphic information content for each locus (PIC), observed heterozygosity (H_O_), expected heterozygosity (H_E_), Shannon’s information index (I), Hardy–Weinberg equilibrium and null allele frequencies, using Genetix v4.05 [21], FSTAT v2.9.3.2 [22], POPGENE Version 1.31 [23] and GenAlEx Version 6.5 [24]. Wright’s F statistics (F_ST_, F_IS_ and FIT), as proposed by Weir and Cockerham [25], were analyzed by using Genetix^®^software [21]. Nei’s gene diversity (H_T_), diversity within populations (H_S_), diversity between populations (D_ST_) and coefficient of gene differentiation (G_ST_) values were analyzed with FSTAT v2.9.4 [22]. Exact tests for deviation from the Hardy–Weinberg Equilibrium (HWE) and partitioning of genetic diversity using analysis of molecular variance (AMOVA) were analyzed, using the ARLEQUIN v. 3.5.2.2 [26]. Pairwise genetic distances (Reynold’s genetic distance) and Nei’s unbiased D_AS_ genetic distances (Nei, [27]) were calculated by using the Populations v 1.2.30 software. Neighbor-net dendrogram constructed from Reynold’s genetic distances by using SplitsTree v4.16.0 [28]. Principal components analysis (PCA) was calculated for the 20 microsatellites, using NTSYSpc V2.10q software [29]. Genetic diversity and the degree of admixture of Turkish water buffalo populations were analyzed by using the Bayesian clustering procedure of STRUCTURE ver. 2.3 [30]. Twenty replicate runs were calculated for each K between 1 and 20, with a burn-in period of 1,000,000 iterations, followed by 500,000 iterations of the Markov chain Monte Carlo algorithm. To determine the most possible groups (K) that best fit the data; we used the STRUCTURE HARVESTER [31], which implements the Evanno method [32]. Evanno’s method was carried out to determine the suitable number of clusters using ∆K, due to the rate of change in the log probability of the data. The program CLUMPP ver. 1.1 [33] was used to align the 20 repetitions of each K. CLUMPP software, an online web-based program, was performed for collating the outcomes produced by the program STRUCTURE. The clustering pattern was applied in the CLUMPP program and visualized by way of the software DISTRUCT software version 1.1 [34].

## 3. Results

In the present study, 20 microsatellite markers were used to analyze the relationships within and among 17 Turkish water buffalo populations. A total of 837 individuals were sampled from 17 provinces, 37 districts and 119 villages belonging to six geographical regions that represent the most important sites of water buffalo breeding.

### Genetic Diversity

A summary of statistic results for genetic diversity is shown in Table 1 and Supplementary Table S2. The mean number of alleles (N_a_), the number of effective alleles (N_e_), observed (H_O_) and unbiased expected (H_E_) heterozygosity, Shannon’s information index (I) and the deficit of heterozygotes (F_IS_) values for individual subpopulations and overall population are presented in Table 1. The properties of the analyzed microsatellite, along with the genetic variation statistics, were listed in Supplementary Table S2. For the entire 17 Turkish water buffalo populations, a total of 190 alleles were found in 837 animal genotypes for the 20 microsatellite markers.

The mean number of alleles (N_a_) was the highest in Central Anatolia (Kayseri (KAY) and Sivas (SVS)) Region buffalo individuals (8.80) and the lowest in Black Sea Region (Giresun (GIR)) buffalo individuals (5.70). The number of effective alleles per locus (N_e_) and allelic richness (R_s_) values are a measure of the genetic diversity. The effective number of alleles per population (N_e_) showed lower values, which varied from 2.87 (Bursa (BUR)) to 4.14 (Muş (MUS)). Additionally, the R_S_ means ranged from 5.03 to 6.83 in the Marmara, Black Sea and Aegean Regions buffaloes, being lower than that in the Central Anatolia, Eastern Anatolia and Southeastern Anatolia Regions buffaloes. The observed (H_O_) and unbiased expected (H_E_) heterozygosity per population varied from 0.55 (BUR) to 0.70 (MUS) and 0.58 (BUR) to 0.73 (MUS), respectively (Table 1). The H_O_ and H_E_ values in the Marmara, Black Sea and Aegean Regions buffaloes were lower than the Central Anatolia, Eastern Anatolia and Southeastern Anatolia Regions’ buffaloes. Genetic diversity of the Turkish water buffalo population of Central Anatolia, Eastern Anatolia and Southeastern Anatolia Regions was observed to be higher than in other regions (Marmara, Aegean and the Black Sea), but the differences were not statistically significant. It is thought that the reason for this difference may be due to the geographical proximity of the buffalo to the domestication center, the fact that the buffalo breeding in these regions consists of small family businesses and there is no selection in this population. The inbreeding coefficient (F_IS_) within the populations varied between 0.017 and 0.183. Additionally, the SVS and Amasya (AMS) populations have shown that the lowest and highest genetic diversity, respectively. All of the F_IS_ values were determined to be positive and statistically significant (*p* < 0.05, *p* < 0.001) (Table 1). The average F_IS_ value, which describes the excess or deficit of heterozygotes within subpopulations was 0.117 (*p* < 0.05) and therefore different from zero. Shannon’s information index (I) across populations ranged from 1.15 (BUR) to 1.58 (MUS).

When the results are evaluated based on microsatellite loci, a total number of alleles per locus varied from 6 (ILSTS005) to 17 (ETH003), while the mean number of alleles per locus (N_a_) ranged between 4.29 and 12.59 for the same loci (Supplementary Table S2). N_e_ ranged from 1.80 (CSSM033) to 7.74 (CSSM047) for the Turkish water buffalo population based on 20 polymorphic microsatellite loci. The value of allelic richness (R_s_) ranged from 3.99 (ILSTS005) to 12.08 (ETH003), with a mean of 6.87. PIC and the Shannon information index are another measure of genetic variability indicating the informativeness of the assessed loci. The polymorphic information content (PIC) was analyzed for each locus and varied from 0.412 (CSSM033) to 0.859 (CSSM047), which has the highest number of alleles per locus in the current study. A total of 16 of these 20 microsatellite loci had polymorphic information content (PIC) values greater than (0.5), which make them useful in genetic diversity studies. Additionally, four loci (CSSM033, ILSTS005, CSSM032 and CSSM029) showed moderate polymorphism (PIC > 0.40) (Supplementary Table S2). Shannon’s information index (I) across populations ranged from 0.91 (CSSM033) to 2.32 (CSSM045). The value of gene flow (Nm*) between the 17 subpopulations was positive and varied between 2.05 (CSSME070) and 26.87 (CSSM029) for different microsatellite loci. This confirms that Turkish buffalo samples were exchanged between the 17 subpopulations.

The values of fixation indexes (F_IS_, F_ST_ and F_IT_) for the overall populations are given in Supplementary Table S2. Most of the markers had positive values for F_IS_ (Supplementary Table S2), showing a deficiency in heterozygosity. The F_IS_ index was negative for ILSTS005, CSSM036 and CSSM029 markers indicating a high frequency of heterozygotes in these loci. F_ST_ values varied from 0.015 to 0.104, and the average values of F_IS_, F_ST_ and F_IT_ were 0.091, 0.031 and 0.119, accordingly. Mean F_ST_ (0.031) was moderate to low, while H_S_ (0.67) was relatively high.

The observed heterozygosity (H_O_), the expected heterozygosity (H_e_), the coefficient of gene distinction (G_ST_), the D_ST_ value and Nei’s gene diversity index (H_T_) per locus was given in Supplementary Table S2.

The average coefficient of gene distinction (G_ST_) over the 20 loci was 0.030 ± 0.021 (*p* < 0.01). The G_ST_ values for single loci varied from −0.001 for CSSM029 to 0.098 for CSSME070. The gene differential coefficient G_ST_ (3%) indicated that most of the total genetic variation was due to intra-population difference and only a few existed among populations, which implied these Turkish water buffalo populations had relatively less genetic diversity and distinctiveness. Nei’s gene diversity index (H_T_) for loci ranged from 0.44 (CSSM033) to 0.88 (CSSM045), with an average of 0.69.

Eighteen of the 20 microsatellite loci found a highly important departure from Hardy–Weinberg equilibrium (HWE) (*p* < 0.001) in the whole population, whereas the other two loci showed different significant differences (*p* < 0.01, *p* < 0.05) (Supplementary Table S2). But, when considering populations separately, many markers per population were in Hardy-Weinberg disequilibrium (*p* > 0.05). The number of these markers ranged between 5 loci in İstanbul-Çatalca (IST) to 14 loci in Tekirdağ (TEK) and GIR populations. All the studied populations performed highly significant departure (*p* < 0.001, *p* < 0.01, *p* < 0.05) from the Hardy–Weinberg equilibrium when considering all microsatellite loci. This departure and the high positive mean values of F_IS_ (Supplementary Table S2) may remark the presence of heterozygote deficiencies, which could be the consequences of an uncontrolled mating between populations. The presence of null alleles, defined as non-amplifying alleles, because of mutations at PCR priming sites, causes overestimation of both F_ST_ and genetic distance values. The null allele frequencies varied from 0.017 (CSSM036) to 0.241 (BMC1013). We identified only one locus (BMC1013) that had potential null alleles at high frequency (r ≥ 0.2) in at least one breed. It has been observed that the frequencies of other microsatellite loci are lower than (0.20) (Supplementary Table S2).

**Figure 1:**
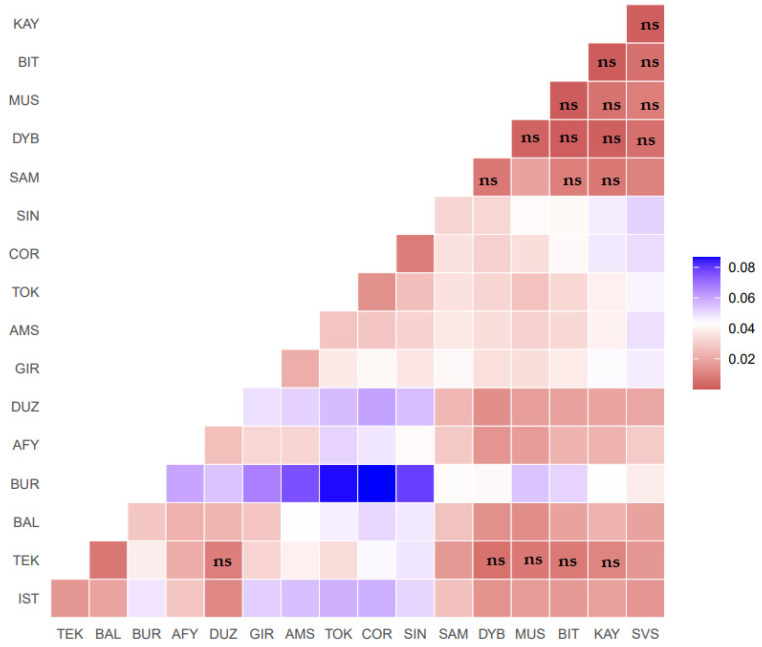
The pairwise F_ST_ distances between the studied 17 Turkish water buffalo populations. Color-codes are identified on the scale at the right side of the figure (ns = not significant, blank significant *p* < 0.001, pairwise populations F_ST_ values and significant were shown in Supplementary Table S3c). İstanbul/Çatalca-IST; Tekirdağ/Saray-TEK; Balıkesir-BAL; Bursa-BUR; Düzce-DUZ; Giresun-GIR; Amasya-AMS; Tokat-TOK; Çorum-COR; Sinop-SIN; Samsun-SAM; Afyon-AFY; Kayseri (KAY), Sivas-SVS; Muş-MUS, Bitlis-BIT; Diyarbakır-DYB.

Population differentiation was compared on the basis of F_ST_, Reynold’s and Nei’s D_AS_ genetic distances in Supplementary Tables S3–S5. The genetic distance of interbreed or F_ST_ values of pairwise comparisons among the 17 Turkish water buffalo populations are given in Figure 1. F_ST_ values of pair-wise comparisons among the 17 populations (the matrix is shown in Supplementary Table S5) of Turkish water buffalo, showed an overall genetic differentiation F_ST_ of (0.032 ± 0.018) and pairwise F_ST_ values ranging from 0.0000 (SVS vs. KAY; KAY vs. Bitlis (BIT)) to 0.0866 (BUR vs. Çorum (COR)) (varying from Indian red, white and blue colors in Figure 1). Significant genetic variation was observed after sequential Bonferroni correction in 118 out of 136 population pairs (Supplementary Table S5).

**Figure 2:**
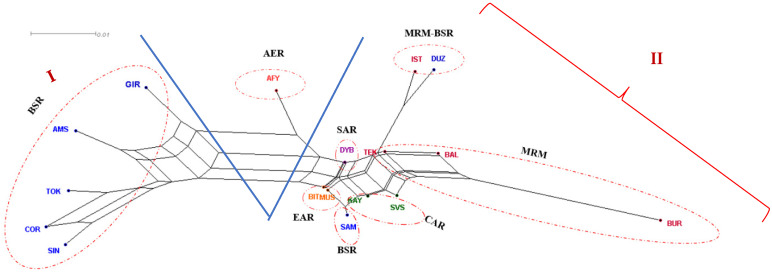
Neighbor-net dendrogram constructed from Reynold’s genetic distances among 17 Turkish water buffalo populations. ^I^ Cluster I; ^II^ Cluster II; Marmara Region (MRM): İstanbul/Çatalca-IST; Tekirdağ/Saray-TEK; Balıkesir-BAL; Bursa-BUR; Black Sea Region (BSR): Düzce-DUZ; Giresun-GIR; Amasya-AMS; Tokat-TOK; Çorum-COR; Sinop-SIN; Samsun-SAM; Aegean Region (AER): Afyon-AFY; Central Anatolian Region (CAR): Kayseri (KAY), Sivas-SVS; Eastern Anatolia Region (EAR): Muş-MUS, Bitlis-BIT; Southeast Anatolian Region (SAR): Diyarbakır-DYB.

The neighbor-net phylogeny performed from Reynold’s genetic distances (Figure 2) visualizes the relations between Turkish water buffalo populations. Populations that shared close genetic relations were located on different branches that originated from the same basal node. It implements two distinct clusters, which are explicitly separated, i.e., (I) from only the Black Sea Region’s buffaloes (GIR, AMS, Tokat (TOK), COR and Sinop (SIN)), (between I and II) from only the Aegean Region’s buffaloes (AFY) and (II) from the Marmara, Black Sea, Central Anatolia, Eastern Anatolia and Southeastern Anatolia Regions’ buffaloes in Turkey (Figure 2). The phylogeny of Reynold’s distances (Supplementary Table S4) was similar to that performed using Nei’s D_AS_ distances (Supplementary Table S3).

The analysis of molecular variance (AMOVA) test was performed to evaluate genetic variability is distributed within and among populations. We calculated possible structures by composing and contrasting different population groups. We performed the analysis under two hypotheses: For Hypothesis (I), the AMOVA analyses results showed that most of the molecular variation occurred within individuals (88.33%), while it represented 0.55% among geographic groups, 2.69% among populations within groups and 8.42% among individuals within populations (Table 2). Variance components among groups, among populations within groups and within individuals were significant (*p* < 0.001) for all the studied loci (Table 2), implementing significant geographical distribution in studied buffalo populations. Furthermore, the variance component among individuals within populations was significant (*p* < 0.05). For Hypothesis (II), AMOVA analyses outcomes indicated that the variation among groups, among populations within groups, among individuals within populations and within individuals was 2.15%, 2.02%, 8.34% and 87.49%, respectively. Variance components among groups, among populations within groups, among individuals within populations and within individuals were significant (*p* < 0.001) for all the studied loci implementing significant Reynold’s genetic distances distribution in studied Turkish water buffalo populations (Table 2).

**Figure 3:**
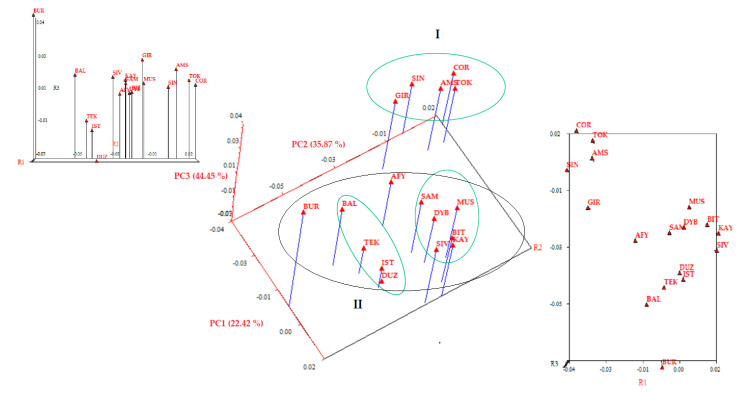
Tree-dimensional principal component analysis (PCA) plot of Turkish water buffalo populations based on 20 microsatellite data. ^I^ Cluster I; ^II^ Cluster II; Marmara Region (MRM): İstanbul/Çatalca-IST; Tekirdağ/Saray-TEK; Balıkesir-BAL; Bursa-BUR; Black Sea Region (BSR): Düzce-DUZ; Giresun-GIR; Amasya-AMS; Tokat-TOK; Çorum-COR; Sinop-SIN; Samsun-SAM; Aegean Region (AER): Afyon-AFY; Central Anatolian Region (CAR): Kayseri (KAY), Sivas-SVS; Eastern Anatolia Region (EAR): Muş-MUS, Bitlis-BIT; South East Anatolian Region (SAR): Diyarbakır-DYB.

The principal component analysis (PCA) of 17 Turkish water buffalo populations due to 20 microsatellite markers is shown in Figure 3. The 44.46% of the total genetic variation present between the populations was explained by the first three axes of the PCA test. This is an acceptable fit, given the small amount of variability from a large number of samples and microsatellite alleles used in the analysis. The PCA analysis classified Turkish water buffalo populations into two basic cluster involving different region populations to some extent, i.e., Cluster I is BSR buffaloes (GIR, SIN, AMS, TOK and COR); Cluster II is the first group, namely BSR, CAR, EAR and SAR buffaloes (Samsun (SAM), DYB, MUS, BIT, SIV and KAY), and the second group, namely MRM and BSR buffaloes (BAL, TEK, IST and Düzce (DUZ)) (Figure 3). The Aegean Region’s buffaloes (AFY) are between the II and III groups. The first component (PC1), which was responsible for 22.42% of the genetic variation, separated the BUR population from all the other studied populations. The second component, which represented 35.87% of the genetic variations, separated the DUZ population (Figure 3).

**Figure 4:**
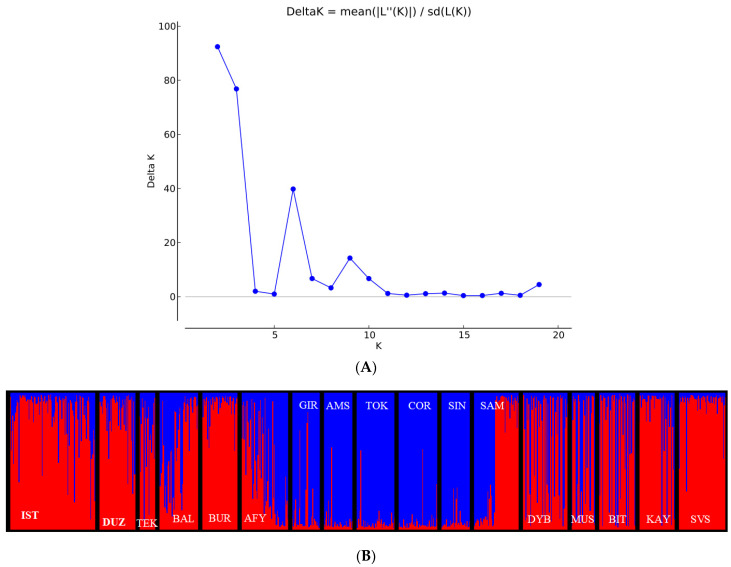
(**A**) Plot of (∆K) values for each K from 1 to 20. (**B**) Clustering analysis by structure for the full-loci dataset assuming K = 2. Blue: Cluster I; Red: Cluster II; Population name abbreviations are labeled below the structure result (Marmara Region (MRM): İstanbul/Çatalca—IST; Tekirdağ/Saray—TEK; Balıkesir—BAL; Bursa—BUR; Black Sea Region (BSR): Düzce—DUZ; Giresun—GIR; Amasya—AMS; Tokat—TOK; Çorum—COR; Sinop—SIN; Samsun—SAM; Aegean Region (AER): Afyon—AFY; Central Anatolian Region (CAR): Kayseri (KAY), Sivas—SVS; Eastern Anatolia Region (EAR): Muş—MUS, Bitlis—BIT; South East Anatolian Region (SAR): Diyarbakır—DYB).

The genetic structure of each population was identified regarding admixture level for each water buffalo individual using a correlated allele frequencies model implemented within the STRUCTURE software. The results of Delta K (∆K = 92.42) represented that the optimal number of genetic clusters indicating most like ancestral breeds was at K = 2 (Figure 4A). The value indicates that the studied water buffalo populations were better identified by two genetic clusters instead of 17 populations (Figure 4B). The two clusters’ backgrounds were made up of IST, DUZ, TEK, Balıkesir (BAL), BUR, SAM, DYB, MUS, BIT, KAY and SVS in the first (red color), and AFY, GIR, AMS, TOK, COR and SIN in the second (dark blue color) cluster (Figure 4B). Approximately 51% of the individuals were classified within their source cluster assuming a threshold of q ≥ 0.700, whereas, for more stringent threshold q values, only ~38% (q ≥ 0.900), ~24% (q ≥ 0.950) and 0.23% (q ≥ 0.999) of the individuals were correctly assigned. There were several heterogeneous populations (AFY, SAM and BAL) with less than 48–30% of the individuals assigned to their source cluster (for q ≥ 0.700). Consequently, the SAM population evidenced two clusters more than an intermediate position between the two reference populations.

**Hypothesis** **I:** *Populations were assigned according to geographical distribution into six groups: (1) MRM = IST, TEK, BAL and BUR; (2) BSR = DUZ, GIR, AMS, TOK, COR, SIN and SAM; (3) AER = AFY; (4) CAR = KAY and SVS; (5) EAR = MUS and BIT; and (6) SAR = DYB.*

**Hypothesis** **II:** *Populations were assigned according to neighbor-net dendrogram constructed from Reynold’s genetic distances distribution into three groups: (I) BSR = GIR, AMS, TOK, COR and SIN; (II) AER = AFY; and (III) IST, DUZ, BAL, BUR, DIY, TEK, SVS, KAY, SAM, BIT and MUS.*

## 4. Discussion

The studies of genetic diversity play a significant role in developing breeding strategies and programs for livestock. In order to retain genetic variation, breeding strategies that increase effective population size minimizing the genetic drift effect should be performed. Microsatellite markers in combination with recent scientific applications of statistic showed a powerful tool for the conservation of native breeds [35].

The advantage of using microsatellite DNA polymorphism to estimate genetic diversity among breed and among associated populations has been researched in livestock such as water buffalo [8,36,37,38,39,40,41,42], cattle [43,44,45,46], sheep [47,48,49] and goat [35,50,51]. To determine genetic diversity among water buffalo populations, several microsatellite studies have been published [12,17,18] in Turkey, but a study of genetic diversity of Turkish water buffalo that has collected 837 individuals from six geographical regions has not been performed until now. In the present study, we wanted to provide basic data on the genetic variation and population structure of Turkish water buffalo populations, using 20 microsatellite markers to provide a foundation for a more comprehensive genetic resource protection and genetic management.

### 4.1. Genetic Diversity of Turkish Water Buffalo

All measures of genetic diversity: the mean number of alleles (N_a_), the number of effective alleles (N_e_), allelic richness (R_s_), Shannon’s Information Index (I) and polymorphic information content values (PIC) showed that most of the studied loci were highly informative, indicating high polymorphism across the loci, thus suggesting the suitability of these microsatellite markers for genetic diversity studies in Turkish water buffalo populations. The values of genetic diversity parameters were higher compared with a similar study of Asian water buffalo population (Pakistan, Thai, Indonesia, Egypt and China) [42,52,53,54,55], South American buffalo populations (Brazil and Colombia) [10,38,56], North American buffalo populations (Cuba) [36,56,57], southeast of Central Europe (Romania) [58] and Southern Europe water buffalo populations (Italia and Greece) [41]. Molecular genetic parameters (N_a_, N_e_, PIC and HO) obtained from this study were higher than previous research in Turkish water buffalo [12,17,18]. These different studies, both the loci and the number of loci involved were different from each other. Thus, differences in the results may partly be attributable to the differences in the loci employed. These results indicated that microsatellites used in the present study have a high confidence to reveal genetic diversity for Turkish water buffalo populations.

The allele diversity for Turkish water buffalo populations was lower than that of the Colombian buffalo population studied with 10 microsatellite loci [10] but much higher than that reported for Cuban, Pakistan, Romanian, Egyptian, Iranian and Brazilian water buffalo populations [52,56,58,59,60]. Turkish water buffalo populations showed a drastic low number of the effective number of alleles (even lower than half) than the observed mean number of alleles. This is due to the very low frequency of most of the alleles at each locus and a very few alleles might have contributed a major part of the allelic frequency at each locus. Allelic richness was considerably high in the CAR’s, EAR’s and SAR’s buffaloes, indicating high genetic polymorphism as expected heterozygosity (>0.6912).

Another measure of genetic variability is expected heterozygosity where the maximum expected heterozygosity (0.7280) was showed in the MUS population and the minimum (0.5833) was shown in the BUR population. This study results indicate the 17 tested Turkish water buffalo population have substantial amount of genetic diversity, when compared to some other water buffalo breeds around the world where, Pakistan water buffalo breeds such as, Nili (H_E_ = 0.53), Ravi(H_E_ = 0.55), Nili-Ravi (H_E_ = 0.54), Kundhi (H_E_ = 0.45), Azi-Kheli (H_E_ = 0.44) [52], Romanian buffalo (H_E_ = 0.4048; Popa et al. [58]), Egyptian buffalo (H_E_ = 0.527; Rushdi et al. [60]); Cuban and Brazilian buffaloes such as, Brazilian Murrah (H_E_ = 0.649), Brazilian Jaffarabadi (H_E_ = 0.643), Cuban Buffalypso/Carabao hybrid (H_E_ = 0.599) [56], Indonesian swamp buffalo (H_E_ = 0.44; Saputra et al. [53]), Thai swamp buffalo (H_E_ = 0.61, Sraphet et al. [54]), Cuban water buffalo population (H_E_ = 0.54, Acosta et al. [57]; H_E_ = 0.509, Uffo et al. [36]), Italian and Greek buffalo populations (H_E_ respectively; 0.57 and 0.59; Moioli et al. [41]), Brazilian buffaloes (H_E_ = 0.558; Marques et al. [38]), Colombian buffalo (H_E_ = 0.70; Ángel-Marín et al. [10]), Guilan buffalo populations (H_E_ = 0.67; Aminafshar et al. [14]), some of Turkish water buffalo populations (H_E_ = 0.5359; Özkan Ünal et al. [12]), Asian swamp buffalo (H_E_ = 0.50; Barker et al. [42]) and Chinese buffalo (H_E_ = 0.53, Zhang et al. [55]). On the other hand, the buffalo populations tested in our study showed less genetic diversity when compared to Iranian indigenous buffalo populations (H_E_ = 0.75; Darestani et al. [59]) and Iraqi buffalo populations (H_E_ = 0.86; Jaayid and Dragh, [61]), Indian river buffalo breeds (H_E_ = 0.71–0.78; Kumar et al. [39]), and African buffalo (H_E_ = 0.759; Van Hooft et al. [62]).

The estimate of inbreeding value (F_IS_) shows the excess of homozygosity in a subpopulation and, with reference to molecular markers, informs if a pattern of reduction in diversity based on several causes exists. The positive F_IS_ values showed heterozygotes deficiency within populations. This deficit might be because of inbreeding and the Wahlund effect. The mean F_IS_ value of the Turkish water buffalo populations was 0.1170 (*p* < 0.05) and is similar to that obtained by Uffo et al. [36] in Cuban water buffalos, but lower than what was described by Ángel-Marín et al. [10], using 10 microsatellite markers in Colombian buffalo herds; other authors describe a lower value of F_IS_ [56,57,58,59]. The F_IS_ in Turkish water buffalo can be considered higher compared with other populations (F_IS_ = 0.1170) like the results reported by Shokrollahi et al. [9], who found a value of 0.047 in the Iranian river buffalo. The highly significant (*p* < 0.001) F_IS_ value (0.1820) showed a rather high inbreeding degree within the population. The heterozygote deficiency performed in the AMS population could be because of the higher rate of inbreeding, the population subdivision (Wahlund effect) and the presence of “null alleles” (non-amplifying alleles). Another possible reason for the high values of inbreeding could be due to their small population size, a small number of breeding males and their limited geographical area of dispersion [63].

The F_IT_ values, which measure the heterozygosity loss of the individual concerning the overall population, were 0.119 (*p* < 0.05), indicating that there is a general lack of heterozygous individuals in the Turkish water buffalo populations of 12%. In the study that the F_IT_ value was considerably lower than the Pakistan buffalo populations [52], Cuban water buffalo breed [36] and Turkish water buffalo populations [17,18], and higher than the values reported by Popa et al. [58], Darestani et al. [59], Marrero et al. [56] and Acosta et al. [57].

The presence of null alleles, defined as non-amplifying alleles, due to mutations at PCR priming sites, causes overestimation of both F_ST_ and genetic distance values. The null allele frequencies in the studied microsatellite loci were below 20% except for BMC1013 loci (24.09%). The lowest and highest null allele frequencies were 0.168 (CSSM036) and 0.2409 (BMC1013), respectively. Taking this value into consideration, it has been implemented that the studied 19 loci can be safely used in paternity tests (except for BMC1013 loci).

PIC value is a parameter indicative of the degree of in formativeness of a microsatellite, may range from 0 to 1. PIC values calculated in this study were comparable with those revealed by Popa et al. [58] in the Romanian buffalo population (0.4335–0.632), Saputra et al. [53] in the Indonesian swamp buffalo population (0.360–0.740), Merdan et al. [64] in five Egyptian buffalo populations (0.65–0.92), Darestani et al. [59] in Iranian indigenous buffalo populations (0.33–0.86), Uffo et al. [36] in Cuban water buffalo populations (0.169–0.809), Marrero et al. [56] in Cuban and Brazilian buffaloes (0.321–0.703), Unal et al. [12] in Turkish water buffalo population (0.14–0.82), Acosta et al. [57] in Cuban water buffalo population (0.201–0.777), Jaayid and Dragh [61] in Iraqi buffalo population (0.11–0.80) and by Ángel-Marín et al. [10] in Colombian water buffalo herds (0.43–0.81). Moreover, results of many studies on Indian and Guilan buffalo breeds revealed also high values of PIC (0.52–0.88) for most studied markers [14,15,65,66]. Other researchers [10,12,67,68,69,70,71,72] reported varied values of PIC for several markers which ranging between moderate (0.34–0.48) and high (0.51–0.82). The PIC values variability observed in the literature may be due to different microsatellite markers used in the studied populations. Our results showed that the high PIC values show that the microsatellite markers used are highly polymorphic and can be useful for analyze the genetic variation in Turkish water buffalo populations.

Mean value of DST indicating genetic diversity between populations, GST, which is an important indicator of the relative magnitude of genetic differentiation, and HT, described as total genetic diversity, values were found as 0.021, 0.030 and 0.694, respectively. The D_ST_ value obtained from this study can be considered as an indicator of low genetic diversity between 17 Turkish water buffalo populations. The average G_ST_ value obtained from the overall loci pointed out that 3% of total genetic diversity resulted from the differences between the populations. In all other respects, it can be said that 97% of genetic variation is caused by the difference between individuals. The G_ST_ values were considerably lower than Ángel-Marín et al. [10] in Colombian buffalo herds. All studied loci showed a significant deviation from the Hardy–Weinberg Equation (*p* > 0.05, *p* > 0.01, *p* > 0.001).

### 4.2. Genetic Structure of Turkish Water Buffalo

Population differentiation was analyzed by estimation of the F_ST_ index. In regard to all pairwise differences (Slatkins linearized F_ST_) in this study, the distribution of F_ST_ represented low genetic divergence (0.000 < F_ST_ < 0.0866) among populations in general. The F_ST_ comparison values obtained were significant in 118 pairwise calculations (*p* < 0.05; *p* < 0.01; *p* < 0.001). The highest level of differentiation was obtained between COR–BUR and TOK–BUR populations (F_ST_ > 0.0866), and the lowest between KAY–BIT and KAY–SVS populations (F_ST_ = 0.000), respectively. Thus, the average proportion of genetic differentiation among breeds was 3.2%. This value is lower than the 16.8% found in another genetic study on Asian buffalo [42], Indian buffalo (3.4%) [39], Turkish water buffalo populations (6.2%) [17], Cuban and Brazilian buffaloes (7.5%) [56] and Pakistan buffalo (7%) [52], but higher than Chinese buffalo (2.8%) [16] and Iranian buffalo (1%) [59]. In the study, it was shown that the F_ST_ value differences between the buffalo populations belonging to the provinces that are geographically close to each other are quite low but important. The reason for this is thought to be due to the male bull changes between nearby provinces.

Neighbor-net representing the Reynolds distance confirmed F_ST_ index findings; AFY population clustered in an intermediate position between the Black Sea Region’s buffaloes (I: SIN, COR, TOK, AMS and GIR) (Cluster I) and another branch (III) formed by Marmara (IST, TEK, BAL and BUR), BSR (DUZ, SAM), Eastern Anatolia (MUS and BIT), Southeastern Anatolia (Diyarbakır (DYB)) and Central Anatolia (KAY and SVS) Regions’ buffaloes. Cluster II: we can obtain the nearest position at the center of the admixture network, indicating a lower genetic distance of TEK, DYB, BAL, SVS, KAY, MUS and BIT with the cluster comprising IST, DUZ and BUR populations. The results of the PCA are concordance with the neighbor-net representing the Reynolds distance network obtained in the present study (Figure 3), with the first three components accounting for only 44.46% of the total variation among the populations.

An AMOVA was implemented to analyze the relative contribution of different factors to the observed genetic variation, with each factor considered in a separate analysis, i.e., six groups according to the geographical prevalence (MRM, BSR, AER, CAR, EAR and SAR), three groups according to Reynold’s genetic distances distribution: The first group is the BSR populations (GIR, AMS, TOK, COR and SIN); the second group is the AER population (AFY); third group comprises the MRM populations (IST, TEK, BAL and BUR), BSR buffaloes (SAM, DUZ), CAR buffaloes (KAY, SVS), EAR buffaloes (MUS, BIT) and SAR buffaloes (DYB). The AMOVA analysis data implemented that the majority of the obtained variance is because of differences among individuals within populations. Most of the variation is obtained within the individuals (88.33% Hypothesis I and 87.49% Hypothesis II), yet the differences among groups show only 0.55% and 2.15% of the variation, respectively. Among groups, among populations within groups and among individuals within populations were also an important origin of variation (*p* < 0.001, *p* < 0.05), though fundamentally smaller than the within individual’s component.

In this study, the analysis with the STRUCTURE program showed that Turkish water buffalo populations were grouped into two major lineages when K = 2 (Figure 4B). Cluster I included IST, BAL, BUR, TEK, DUZ, SVS, KAY, BIT, MUS and DYB populations, and Cluster II gathered GIR, AMS, TOK, COR and SIN populations, while other water buffalo populations (AFY and SAM) appeared to be the contact zone between both clusters, as individuals had mixed lineages. The STRUCTURE analysis results support the neighbor-net dendrogram results, as well as F_ST_, PCA and genetic distance results. Our results provide a broad perspective on the extant genetic variation and population structure of Turkish water buffalo populations.

Identification of within and between populations genetic diversity is a prerequisite for well-structured and sustainable animal breeding and conservation programs. The study, which was carried out by using 20 microsatellite markers, showed that within population genetic diversity was higher than between population diversity. This situation can be seen as an opportunity in terms of breeding programs and genetic conservation programs for these populations. Hence, it can be concluded that the Turkish water buffalo population possessed a considerable amount of genetic diversity due to the low pressure of artificial selection and the possibility of random mating; however, Turkish buffalo populations require a scientific production system in order to improve the production, without losing the significant genetic structure of these economically important animals.

## 5. Conclusions

In this paper, we have provided noticeably powerful data on genetic diversity and population structure of Turkish water buffalo populations, which might be helpful for similar studies. Our results suggested the relatively low but statistically significant genetic diversity of 17 Turkish water buffalo populations and brought insight into the structure of the analyzed populations. This study is the first assessment of the molecular genetic diversity of six geographical regions’ water buffalo populations in Turkey. The findings on the genetic structure of Turkish water buffalo populations in the present study will have significant implications in formulating the future strategies for conservation and breeding programs.

## Figures and Tables

**Table 1:** Genetic diversity of populations of Turkish water buffalo populations. The number of individuals (N), the mean number of alleles (N_a_), the number of effective alleles (N_e_), allelic richness (R_s_), observed heterozygosity (H_O_), unbiased expected heterozygosity (H_E_), Shannon’s information index (I) and deficit of heterozygotes (F_IS_).

Region	Location	Latitude/Longitude	N	Na	N	Rs	H_O_	H_E_	I	F_IS_
	İstanbul–Çatalca (IST)	41°06′ N 28°30′ E	104	8.30	3.58	6.21	0.59	0.67	1.42	0.114 ***
Marmara Region (MRM)	Tekirdağ (TEK)	41°29′ N 27°59′ E	23	5.85	3.46	5.81	0.63	0.68	1.35	0.081 *
	Balıkesir (BAL)	39°39′ N 27°53′ E	50	6.75	3.34	5.83	0.61	0.66	1.33	0.068 *
	Bursa (BUR)	40°11′ N 29°04′ E	46	6.00	2.87	5.03	0.55	0.58	1.15	0.055 *
Black Sea Region (BSR)	Düzce (DUZ)	40°49′ N 31°10′ E	47	7.00	3.37	5.36	0.59	0.67	1.38	0.111 ***
	Giresun (GIR)	49°55′ N 38°24′ E	37	5.70	3.27	5.77	0.58	0.65	1.28	0.095 ***
	Amasya (AMS)	40°39′ N 35°51′ E	38	6.30	3.64	5.83	0.55	0.67	1.37	0.182 ***
	Tokat (TOK)	40°19′ N 36°43′ E	49	6.50	3.47	5.73	0.59	0.68	1.38	0.127 ***
	Çorum (COR)	39°14′ N 38°27′ E	50	6.65	3.55	5.49	0.58	0.67	1.36	0.119 ***
	Sinop (SIN)	42°01′ N 35°09′ E	38	6.00	3.21	6.83	0.56	0.64	1.28	0.126 ***
	Samsun (SAM)	41°17′ N 36°20′ E	57	8.35	3.60	5.90	0.61	0.65	1.43	0.071 ***
Aegean Region (AER)	Afyon (AFY)	38°45′ N 30°33′ E	59	7.15	3.49	6.01	0.58	0.67	1.38	0.135 ***
Central Anatolia Region (CAR)	Kayseri (KAY)	38°43′ N 35°30′ E	46	8.80	3.84	7.43	0.69	0.70	1.55	0.023 ^NS^
	Sivas (SVS)	39°45′ N 37°02′ E	57	8.80	3.82	7.22	0.68	0.69	1.52	0.014 ^NS^
Eastern Anatolia Region (EAR)	Muş (MUS)	38°44′ N 41°30′ E	32	8.20	4.14	7.20	0.70	0.73	1.58	0.029 ^NS^
	Bitlis (BIT)	38°22′ N 42°06′ E	47	8.75	4.03	7.53	0.67	0.72	1.58	0.063 ***
Southeastern Anatolia Region (SAR)	Diyarbakır (DYB)	37°18′ N 40°44′ E	57	8.60	4.00	7.52	0.66	0.71	1.55	0.057 ***
Overall ^a^/Mean			837	7.27	3.57	6.87	0.61	0.70	1.41	0.117 *

^NS^ Not significant, * *p* < 0.05; *** *p* < 0.001, Marmara Region (MRM): İstanbul/Çatalca- IST; Tekirdağ / Saray-TEK; Balıkesir-BAL; Bursa—BUR; Black Sea Region (BSR): Düzce-DUZ; Giresun-GIR; Amasya—AMS; Tokat-TOK; Çorum-COR; Sinop-SIN; Samsun—SAM; Aegean Region (AER): Afyon-AFY; Central Anatolian Region (CAR): Kayseri (KAY), Sivas-SVS; Eastern Anatolia Region (EAR): Muş-MUS, Bitlis-BIT; South East Anatolian Region (SAR): Diyarbakır-DYB.

**Table 2:** Analysis of molecular variance (AMOVA) results for 17 populations of Turkish buffalo based on 20 microsatellite data, using the ARLEQUIN program.

Source of Variation	Variance Component (Estimate)	Variance (%)	Fixation Index	*p*-Value ^a^
	Hypothesis I: Geographical distribution			
Among groups	0.0379 (V_a_)	0.55	Φ_IS_:0.0871	0.0000 ^***^
Among populations within groups	0.1856 (V_b_)	2.69	Φ_SC_:0.0271	0.0000 ^***^
Among individuals within populations	0.5809 (V_c_)	8.42	Φ_CT_:0.0055	0.0938 ^*^
Within individuals	6.0908 (V_d_)	88.33	Φ_IT_:0.1167	0.0000 ^***^
	Hypothesis II: Neighbor-net dendrogram constructed from Reynold’s genetic distances distribution			
Among groups	0.14957 (V_a_)	2.15	Φ_IS_:0.08707	0.0000 ^***^
Among populations within groups	0.14033 (V_b_)	2.02	Φ_SC_:0.02060	0.0000 ^***^
Among individuals within populations	0.58091 (V_c_)	8.34	Φ_CT_:0.02148	0.0000 ^***^
Within individuals	6.09080 (V_d_)	87.49	Φ_IT_:0.12509	0.0000 ^***^

^a^: * *p* < 0.05; *** *p* < 0.001.

## Data Availability

The data that support the findings of this study are available from the corresponding author (E.Ö.Ü.), upon reasonable request.

## Supplementary Materials

The following are available online at https://www.mdpi.com/article/10.3390/ani11041067/s1. Table S1: Information about the microsatellite loci used for genetic diversity analyses of Turkish water buffalo populations. Table S2. Genetic polymorphism parameters estimated for 20 microsatellite markers over all Turkish water buffalo populations. Table S3. DAS genetic distance; Table S4. Reynold’s genetic distance values. Table S5. The pairwise FST distance values between the studied 17 Turkish water buffalo populations, Figure S1. Geographic distribution of the 17 Turkish water buffalo populations included in the study.
