# Supplementary material for: Evaluation of Genetic Diversity and Structure of Turkish Water Buffalo Population by Using 20 Microsatellite Markers
Source: Animals (Basel). 2021 Apr 9;11(4):1067. doi: 10.3390/ani11041067 (PMC8070036; doi:10.3390/ani11041067)
Supplement: Supplementary file 1 [file animals-11-01067-s001.pdf]

animals

MDPI

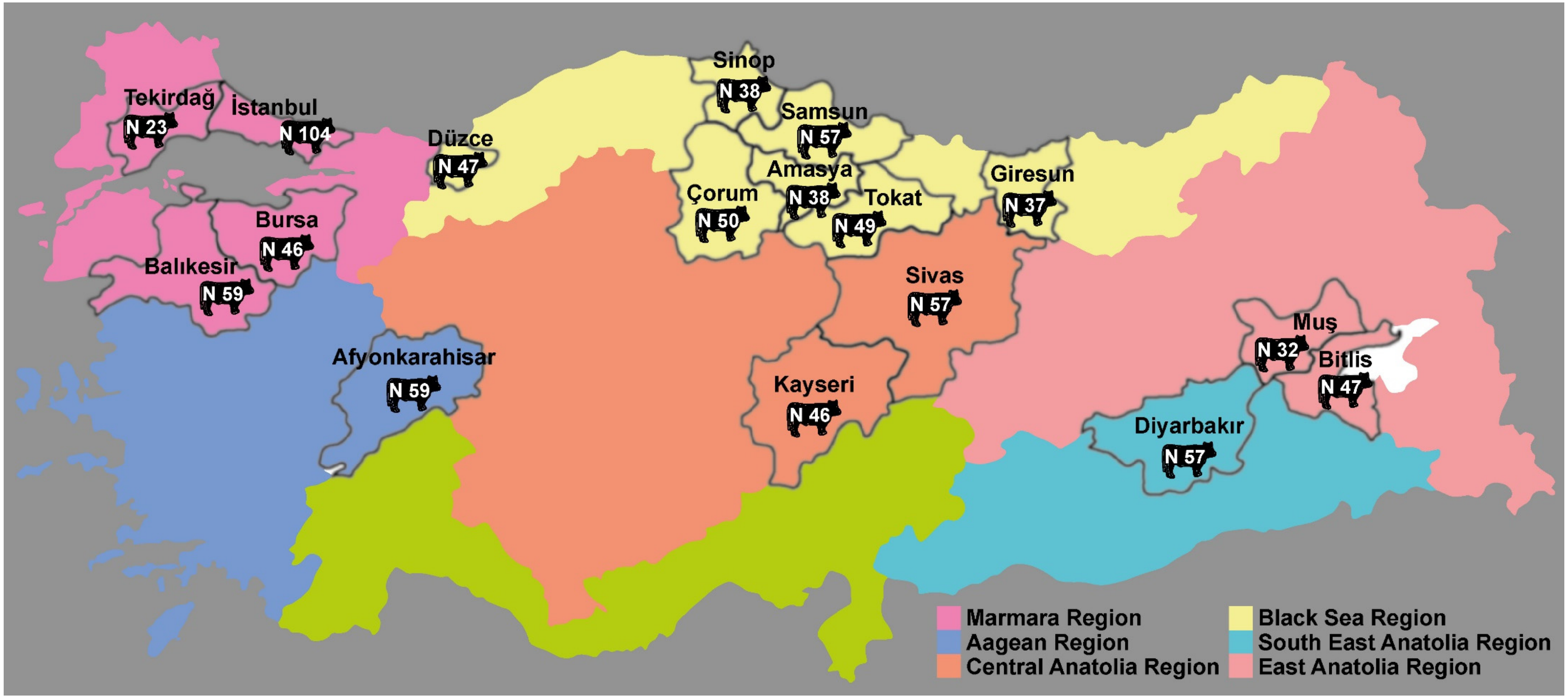

Supplementary Figure S1. Geographic distribution of the 17 Turkish water buffalo populations included in the study. n=number of samples from each province. MRM: Marmara Region, AER: Aagean Region, CAR: Central Anatolia Region, BSR: Black Sea Region, SAR: South East Anatolia Region, EAR: East Anatolia Region.

Supplementary Table S1. Information about the microsatellite loci used for genetic diversity analyses of Turkish water buffalo populations.

Name(s)	Chromosome[1]	Primer sequence (5' → 3')<br>Forward / Reverse	Annealing Temperature (°C)	GenBank (Accession Number) or citation	Allele range (bp)	Multiplex Group (Dye)	Repetitive sequence	Allele range (bp)
**CSSM061**	8	AGGCCATATAGGAGGCAAGCTTAC<br>TTCAGAAGAGGGCAGAGAATACAC	60	Barker et al.. 1997	100-126	1 (HEX)	-	99-125
**CSSM033**	17 (17)	CACTGTGAATGCATGTGTGTGAGC<br>CCCATGATAAGAGTGCAGATGACT	65	U03805	154-175	1(FAM)	$(GT)_n$	151-171
**ILSTS005**	11 (10)	GGAAGCAATGAAATCTATAGCC<br>TGTTCTGTGAGTTTGTAAGC	55	L23481	173-186	1(HEX)	$(TG)_n$	173-189
**CSSM022**	4q (5)	TCTCTCTAATGGAGTTGGTTTTTG<br>ATATCCCACTGAGGATAAGAATTC	55-60	U03806	203-213	1(FAM)	$(CA)_n$	245-267
**CSRM060**	11 (10)	AAGATGTGATCCAAGAGAGAGGCA<br>AGGACCAGATCGTGAAAGGCATAG	60	AF232758	95-135	2 (FAM)	$(AC)_n$	91-133
**BRN**	11 (10)	CCTCCACACAGGCTTCTCTGACTT<br>CCTAACTTGCTTGAGTTATTGCCC	60	X59767	121-147	2 (HEX)	$(AC)_n$	121-147
**ILSTS033**	13 (12)	TATTAGAGTGGCTCAGTGCC<br>ATGCAGACAGTTTTAGAGGG	55	L37213	126-138	2 (TAMRA)	$(AC)_{11}$	137-157
**CSSM032**	1q (1)	TTATTTTCAGTGTTTCTAGAAAAC<br>TATAATATTGCTATCTGGAAATCC	55	U03811	208-224	2(FAM)	$(CA)_n$	235-265
**CSSM045**	2q (2)	TAGAGGCACAAGCAAACCTAACAC<br>TTGGAAAGATGCAGTAGAACTCAT	60	U03830	102-122	3(HEX)	(CA)n	99-125
**CSSM062**	9	GTTTAAACCCCAGATTCTCCCTTG<br>AGATGTAACAGCATCATGACTGAA	55	(Barker et al. 1997)	124-136	3(FAM)	-	113-131
**ILSTS030**	2q (2)	CTGCAGTTCTGCATATGTGG<br>CTTAGACAACAGGGGTTTGG	55	L37212	146-158	3(HEX)	$(GT)_n$	153-169
**BMC1013**	3p (19)	AAAAATGATGCCAACCAAATT<br>TAGGTAGTGTTCCTTATTTCTCTGG	54	G18560	217-239	3(FAM)	$(CA)_n$	292-314
**CSSM057**	9 (7)	GTCGCTGGATAAACAATTTAAAGT<br>TGTGGTGTTTAACCCTTGTAATCT	60	U03840	102-130	4(FAM)	$(TG)_n$	106-128
**CSSME070**	3p (19)	TTCTAACAGCTGTCACTCAGGC<br>ATACAGATTAAATACCCACCTG	50-55	AF303223	119-139	4(TAMRA)	$(AC)_n$	100-134
**CSSM036**	1p (27)	GGATAACTCAACCACACGTCTCTG<br>AAGAAGTACTGGTTGCCAATCGTG	55	U03827	162-176	4(FAM)	$(TG)_n$	149-179
**CSSM043**	1p (27)	AAAACTCTGGGAACTTGAAAACTA<br>GTTACAAATTTAAGAGACAGAGTT	55	U03824	222-258	4(HEX)	$(CA)_n(TC)_n$	173-215
**ETH003**	3p (19)	GAACCTGCCTCTCCTGCATTGG<br>ACTCTGCCTGTGGCCAAGTAGG	65	Z22744	96-192	5(FAM)	$(GT)_n$ AC $(GT)_6$	105-169
**CSSM047**	3q (8)	TCTCTGTCTCTATCACTATATGGC<br>CTGGGCACCTGAAACTATCATCAT	55	U03821	127-162	5(TAMRA)	$(TG)_n$	115-169
**CSSM029**	9 (7)	GCTCCATTATGCACATGCCATGCT<br>CGTGAGAACCGAAAGTCACACATTC	55	U03807	174-196	5(FAM)	$(AC)_n$	161-185
**ETH121**	2q (2)	CCAACTCCTTACAGGAAATGTC<br>ATTTAGAGCTGGCTGGTAAGTG	59	Z14037	182-198	5(HEX)	$(TG)_n$	181-209

[1]Cattle chromosome assignments in parentheses.

animals

MDPI

Supplementary Table S2. Genetic polymorphism parameters estimated for 20 microsatellite markers over all Turkish water buffalo populations.

Loci	TNA	N_a	Ne	Rs	PIC	I	N_m^*	F_{IS}^a	F_{ST}^a	F_{IT}^a	H_O	H_E	H_T	H_S	D_{ST}	G_{ST}	HWE	$F_{(null)}$	SR
**CSSM061**	9	7.71	5.23	7.73	0.787	1.86	7.23	0.028	0.023	0.050	0.77	0.81	0.81	0.79	0.018	0.023	***	0.101	109-125
**CSSM033**	7	4.88	1.80	4.35	0.412	0.91	6.88	0.129	0.028	0.154	0.38	0.44	0.44	0.43	0.010	0.023	***	0.075	151-171
**ILSTS005**	6	4.29	2.30	3.99	0.471	0.98	7.02	-0.089	0.023	-0.064	0.50	0.57	0.56	0.55	0.014	0.025	***	0.029	173-189
**CSSM022**	8	6.71	3.03	6.15	0.628	1.41	9.26	0.116	0.015	0.130	0.58	0.67	0.67	0.66	0.010	0.015	***	0.059	245-267
**CSRM060**	10	7.71	3.65	7.38	0.697	1.64	6.26	0.013	0.028	0.042	0.70	0.73	0.73	0.71	0.020	0.028	**	0.017	91-133
**BRN**	9	6.59	2.49	5.80	0.561	1.27	8.45	0.151	0.017	0.165	0.50	0.60	0.60	0.59	0.010	0.017	***	0.041	121-147
**ILSTS033**	7	5.94	2.84	5.31	0.612	1.33	9.38	0.089	0.015	0.103	0.59	0.65	0.66	0.65	0.010	0.015	***	0.075	137-157
**CSSM032**	8	6.18	1.80	5.64	0.429	1.02	4.18	0.249	0.048	0.286	0.33	0.45	0.46	0.44	0.020	0.044	***	0.151	235-265
**CSSM045**	13	10.88	8.41	11.44	0.869	2.32	4.68	0.221	0.045	0.257	0.67	0.88	0.88	0.85	0.034	0.038	***	0.099	99-125
**CSSM062**	10	7.77	5.66	7.53	0.800	1.88	5.55	0.178	0.037	0.209	0.66	0.82	0.82	0.79	0.025	0.031	***	0.091	113-131
**ILSTS030**	7	5.41	2.55	4.76	0.532	1.14	7.25	0.093	0.019	0.111	0.55	0.61	0.62	0.60	0.014	0.022	***	0.044	153-169
**BMC1013**	9	7.94	4.51	7.36	0.747	1.73	4.00	0.159	0.047	0.199	0.61	0.78	0.77	0.74	0.036	0.047	***	0.241	292-314
**CSSM057**	8	6.24	4.44	5.93	0.737	1.60	9.29	0.049	0.016	0.064	0.73	0.78	0.77	0.76	0.012	0.015	*	0.032	106-128
**CSSME070**	10	7.12	6.12	7.84	0.815	1.94	2.05	0.086	0.104	0.181	0.69	0.84	0.84	0.76	0.083	0.098	***	0.191	100-134
**CSSM036**	10	6.77	2.91	6.01	0.594	1.34	6.99	-0.085	0.024	-0.059	0.70	0.66	0.65	0.64	0.016	0.025	***	0.017	149-179
**CSSM043**	8	5.53	2.57	5.25	0.570	1.25	3.31	0.074	0.060	0.129	0.55	0.61	0.62	0.58	0.037	0.060	***	0.067	173-215
**ETH003**	17	12.59	5.13	12.08	0.789	2.17	8.07	0.217	0.019	0.231	0.61	0.81	0.80	0.79	0.014	0.017	***	0.089	105-169
**CSSM047**	14	11.18	7.74	10.88	0.859	2.26	7.87	0.091	0.021	0.110	0.78	0.87	0.87	0.86	0.017	0.020	***	0.077	115-169
**CSSM029**	10	5.82	2.17	4.52	0.461	1.01	26.87	-0.042	0.023	0.050	0.56	0.54	0.54	0.54	-0.001	-0.001	***	0.030	161-185
**ETH121**	10	8.29	4.16	7.54	0.731	1.72	8.48	0.042	0.020	0.061	0.72	0.76	0.76	0.75	0.014	0.018	***	0.027	181-209
Mean		7.28	3.98	6.87	0.655	1.54	5.85	0.091	0.031	0.119	0.61	0.69	0.69	0.67	0.021	0.030	-	-	-

TNA-Total number of alleles; Na-Mean number of alleles; Ne-Effective number of alleles; PIC-Polymorphic information content for each locus; F statistics (F_{IS}, F_{ST}, F_{IT}); H_O-Observed heterozygosity; H_E-Expected heterozygosity; H_T-Nei's gene diversity; Hs-Diversity within breeds; D_{ST}-diversity between breeds; G_{ST}-coefficient of gene differentiation; HWE-test for significant deviation from Hardy-Weinberg equilibrium with the hypothesis of the heterozygote excess; SR-Size range of the observed allele in bp. a Wright's statistics according to Weir and Cockerham (1984). *P <0.05; **P<0.01, ***p<0.001, Nm* gene flow estimated from Nm=0.25 (1-F_{ST}) / F_{ST} (Nei, 1987).

Supplementary Table S3. D_{AS} genetic distance

	IST	TEK	BAL	BUR	AFY	DUZ	GIR	AMS	TOK	COR	SIN	SAM	DYB	MUS	BIT	KAY	SVS
**IST**	0.0000																
**TEK**	0.0320	0.0000															
**BAL**	0.0406	0.0157	0.0000														
**BUR**	0.0899	0.0711	0.0520	0.0000													
**AFY**	0.0567	0.0424	0.0469	0.1095	0.0000												
**DUZ**	0.0257	0.0165	0.0478	0.1007	0.0584	0.0000											
**GIR**	0.1144	0.0686	0.0629	0.1256	0.0652	0.1058	0.0000										
**AMS**	0.1228	0.0896	0.0972	0.1505	0.0671	0.1184	0.0483	0.0000									
**TOK**	0.1242	0.0708	0.0997	0.1642	0.1038	0.1225	0.0776	0.0616	0.0000								
**COR**	0.1294	0.0956	0.1102	0.1650	0.0959	0.1352	0.0820	0.0594	0.0299	0.0000							
**SIN**	0.1118	0.0993	0.1022	0.1483	0.0849	0.1233	0.0713	0.0651	0.0520	0.0189	0.0000						
**SAM**	0.0618	0.0322	0.0594	0.0828	0.0588	0.0546	0.0813	0.0808	0.0648	0.0701	0.0611	0.0000					
**DYB**	0.0433	0.0165	0.0370	0.0880	0.0369	0.0387	0.0854	0.0904	0.0751	0.0738	0.0793	0.0179	0.0000				
**MUS**	0.0479	0.0233	0.0350	0.1122	0.0465	0.0497	0.0898	0.0891	0.0761	0.0893	0.1069	0.0474	0.0073	0.0000			
**BIT**	0.0464	0.0221	0.0474	0.1100	0.0550	0.0515	0.0898	0.0858	0.0777	0.0951	0.0944	0.0197	-0.00003	-0.0008	0.0000		
**KAY**	0.0526	0.0290	0.0586	0.0933	0.0582	0.0545	0.1061	0.1053	0.0951	0.1111	0.1099	0.0221	0.0041	0.0135	-0.0035	0.0000	
**SVS**	0.0463	0.0394	0.0509	0.0829	0.0725	0.0546	0.1133	0.1259	0.1073	0.1188	0.1229	0.0285	0.0159	0.0229	0.0126	-0.0021	0.0000

Supplementary Table S4. Reynold's genetic distance values.

	IST	TEK	BAL	BUR	AFY	DUZ	GIR	AMS	TOK	COR	SIN	SAM	DYB	MUS	BIT	KAY	SVS
**IST**	0																
**TEK**	0.0179	0															
**BAL**	0.0229	0.0085	0														
**BUR**	0.0565	0.0387	0.0295	0													
**AFY**	0.0331	0.0249	0.0255	0.0698	0												
**DUZ**	0.0133	0.0124	0.0291	0.0622	0.0318	0											
**GIR**	0.0609	0.0359	0.0317	0.0780	0.0378	0.0606	0										
**AMS**	0.0754	0.0508	0.0593	0.1021	0.0404	0.0713	0.0263	0									
**TOK**	0.0704	0.0422	0.0582	0.1096	0.0600	0.0707	0.0428	0.0312	0								
**COR**	0.0748	0.0557	0.0646	0.1108	0.0575	0.0790	0.0461	0.0320	0.0160	0							
**SIN**	0.0653	0.0599	0.0616	0.1022	0.0506	0.0728	0.0408	0.0365	0.0303	0.0115	0						
**SAM**	0.0318	0.0180	0.0328	0.0502	0.0333	0.0298	0.0450	0.0412	0.0397	0.0404	0.0380	0					
**DYB**	0.0166	0.0070	0.0156	0.0438	0.0168	0.0169	0.0386	0.0410	0.0370	0.0364	0.0390	0.0086	0				
**MUS**	0.0182	0.0090	0.0144	0.0575	0.0196	0.0218	0.0386	0.0383	0.0325	0.0412	0.0504	0.0215	0.0033	0			
**BIT**	0.0176	0.0102	0.0227	0.0556	0.0254	0.0220	0.0426	0.0382	0.0371	0.0475	0.0472	0.0093	0.0017	0.0015	0		
**KAY**	0.0208	0.0129	0.0269	0.0448	0.0258	0.0228	0.0497	0.0472	0.0453	0.0549	0.0540	0.0075	0.0024	0.0073	0.0006	0	
**SVS**	0.0177	0.0175	0.0225	0.0405	0.0326	0.0226	0.0526	0.0601	0.0536	0.0585	0.0610	0.0112	0.0063	0.0101	0.0065	-0.0001	0

Supplementary Table S5. The pairwise F_{ST} distance values between the studied 17 Turkish water buffalo populations

	IST	TEK	BAL	BUR	AFY	DUZ	GIR	AMS	TOK	COR	SIN	SAM	DYB	MUS	BIT	KAY	SVS
IST	-																
TEK	0.01567***	-															
BAL	0.01903***	0.00722NS	-														
BUR	0.04832***	0.03866***	0.02857***	-													
AFY	0.02820***	0.02135***	0.02257***	0.06031***	-												
DUZ	0.01137***	0.00934NS	0.02362***	0.05454***	0.02629***	-											
GIR	0.05215***	0.03183***	0.02779***	0.06735***	0.03243***	0.04934***	-										
AMS	0.05550***	0.03935***	0.04380***	0.07604***	0.03223***	0.05187***	0.02184***	-									
TOK	0.05801***	0.03411***	0.04633***	0.08589***	0.05134***	0.05583***	0.03766***	0.02761***	-								
COR	0.05830***	0.04485***	0.05080***	0.08662***	0.04793***	0.06096***	0.04148***	0.02836***	0.01366 ***	-							
SIN	0.05115***	0.04804***	0.04764***	0.07936***	0.04213***	0.05567***	0.03665***	0.03151***	0.02625***	0.00885NS	-						
SAM	0.02667***	0.01608***	0.02715***	0.04232***	0.02870***	0.02409***	0.04132***	0.03730***	0.03525***	0.03549***	0.03240***	-					
DYB	0.01439***	0.00550NS	0.01379***	0.04181***	0.01498***	0.01357***	0.03498***	0.03452***	0.03215***	0.03129***	0.03267***	0.00750NS	-				
MUS	0.01661***	0.00737NS	0.01300***	0.05452***	0.01709***	0.01781***	0.03471***	0.03129***	0.02729***	0.03467***	0.04227***	0.01899 ***	0.00212 NS	-			
BIT	0.01601***	0.00791NS	0.01887***	0.05125***	0.02301***	0.01838***	0.03803***	0.03317***	0.03282***	0.04176***	0.04146***	0.00915 NS	0.00067NS	0.00017NS	-		
KAY	0.01798***	0.01087NS	0.02300***	0.04317***	0.02297***	0.01903***	0.04413***	0.04001***	0.03942***	0.04739***	0.04670***	0.00736 NS	0.00130 NS	0.00585NS	0.00036NS	-	
SVS	0.01509***	0.01559***	0.01880***	0.03869***	0.02973***	0.01956***	0.04668***	0.04911***	0.04543***	0.04957***	0.05156***	0.01030***	0.00537 NS	0.00912NS	0.0053NS	0.00080NS	-

*** Significant at $P < 0.001$.
